# Molecular Regulation of Arterial Aneurysms: Role of Actin Dynamics and microRNAs in Vascular Smooth Muscle

**DOI:** 10.3389/fphys.2017.00569

**Published:** 2017-08-10

**Authors:** Azra Alajbegovic, Johan Holmberg, Sebastian Albinsson

**Affiliations:** Department of Experimental Medical Science, Lund University Lund, Sweden

**Keywords:** microRNA, aneurysm, BAV, actin polymerization, myocardin related transcription factors

## Abstract

Aortic aneurysms are defined as an irreversible increase in arterial diameter by more than 50% relative to the normal vessel diameter. The incidence of aneurysm rupture is about 10 in 100,000 persons per year and ruptured arterial aneurysms inevitably results in serious complications, which are fatal in about 40% of cases. There is also a hereditary component of the disease and dilation of the ascending thoracic aorta is often associated with congenital heart disease such as bicuspid aortic valves (BAV). Furthermore, specific mutations that have been linked to aneurysm affect polymerization of actin filaments. Polymerization of actin is important to maintain a contractile phenotype of smooth muscle cells enabling these cells to resist mechanical stress on the vascular wall caused by the blood pressure according to the law of Laplace. Interestingly, polymerization of actin also promotes smooth muscle specific gene expression via the transcriptional co-activator MRTF, which is translocated to the nucleus when released from monomeric actin. In addition to genes encoding for proteins involved in the contractile machinery, recent studies have revealed that several non-coding microRNAs (miRNAs) are regulated by this mechanism. The importance of these miRNAs for aneurysm development is only beginning to be understood. This review will summarize our current understanding about the influence of smooth muscle miRNAs and actin polymerization for the development of arterial aneurysms.

## Introduction

Aneurysms are caused by a weakening in the arterial wall resulting in a local distension of the affected vessel. Although aneurysms can occur at various sites of the vasculature, aortic aneurysms are the most common and typically classified in terms of their anatomical location: thoracic aortic aneurysms (TAA) and abdominal aortic aneurysms (AAA). A study from the Global Burden of Disease lists aortic aneurysm among the 10 most common causes of cardiovascular disease-related death (Roth et al., 2015).

Aneurysm pathology is characterized by endothelial dysfunction, reduced contractile function due to altered actin dynamics and/or changes in smooth muscle phenotype, and degradation of elastic fibers and collagen. Although the etiology of aneurysms may differ depending on the affected site, certain mechanisms involved in the progressive weakening of the vascular wall are likely general for multiple forms of aneurysms. In most cases, aneurysms develop slowly and cause no noticeable symptoms until rupture occurs, hampering early intervention. There are currently no therapeutic drugs available and current treatment options are limited to open surgery or endovascular repair. Consequently, there is an acute need for additional therapeutic approaches.

According to the National Heart, Lung, and Blood Institute, environmental risk factors for aortic aneurysms include age, male gender, smoking, and high blood pressure. In addition, genetic factors play a role in the cause of aortic aneurysms, albeit stronger for TAA than AAA (Biddinger et al., 1997; Morisaki and Morisaki, 2016). TAA is a common finding in conditions such as Marfan and Loeys-Dietz syndrome (Dietz et al., 1991; Loeys et al., 2006), and bicuspid aortic valve (BAV) (Prakash et al., 2014). BAV is present in 0.5–2% of the population and is the most common congenital heart anomaly although symptoms, including TAA, typically develop in adulthood (Hoffman and Kaplan, 2002; Siu and Silversides, 2010). It is clinically heterogenous and the exact cause is unclear. Unlike Marfan syndrome and Loeys-Dietz syndrome, which is caused by mutations in the *FBN1* and *TGFBR1*/*TGFBR2* genes, respectively, no gene causing BAV has been identified.

Inherited predisposition to thoracic aortic disease in the absence of syndromic features has also been reported. Recent studies demonstrate that mutations in *ACTA2* and *MYH11*, encoding the contractile proteins smooth muscle cell α-actin and β-myosin heavy chain, respectively, can cause thoracic aortic aneurysms and dissections (TAAD) (Zhu et al., 2006; Guo et al., 2007). Importantly, mutations in *ACTA2* are the most prevalent genetic cause of TAAD and to date more than 40 *ACTA2* mutations have been identified (Guo et al., 2007; Morisaki et al., 2009; Regalado et al., 2015). Some of the *ACTA2* mutations have been shown to interfere with actin polymerization (Guo et al., 2007; Malloy et al., 2012; Lu et al., 2015). As such, *ACTA2* mutations may lead to a defective contractile function and reduced ability of vascular smooth muscle cells (SMCs) to resist mechanical stress on the arterial wall and consequently increasing the susceptibility for aneurysm and dissection. The first part of this Review focuses on actin polymerization in formation of aneurysms and its potential role for the regulation of gene expression via the myocardin related transcription factor, MRTF.

Recently, microRNAs (miRNAs) have been associated with the formation of both aortic and intracranial aneurysms. MiRNAs are small, single-stranded non-coding RNAs. They are ~22 nucleotides in length and regulate gene expression post-transcriptionally by binding to complementary target sites in mRNA molecules. A number of miRNAs are differentially expressed in aortic aneurysms, including miR-29 (Boon et al., 2011), members of the miR-15 family (Zampetaki et al., 2014), miR-21 (Maegdefessel et al., 2012a), miR-26 (Leeper et al., 2011), and miR-143/145 (Elia et al., 2009). Moreover, we have identified a group of miRNAs regulated by actin polymerization and MRTF. Several of these miRNAs are downregulated in dilated aorta suggesting that they may play a role in the development of aneurysms (Alajbegovic et al., 2016). In the second part of this Review, we focus on the importance of miRNAs for the formation of arterial aneurysms. Taken together, identification and characterization of both coding and non-coding genes associated with actin polymerization may aid in the development of much needed new therapeutic strategies against aneurysms formation. These may not be specifically involved in BAV-associated aortopathy, but it is clear that common mechanisms are involved in various forms of arterial aneurysms, which can improve our understanding of the cause of this disease.

## The role of actin polymerization in the development of arterial aneurysm

Mutations that have been linked to arterial aneurysm involve dynamic changes in polymerization of actin filaments (Guo et al., 2009; Lu et al., 2015). Actins constitute a family of highly conserved proteins that polymerizes into filaments and play a number of important roles in various biological processes including force generation, cellular mechanosensing, regulation of cell differentiation, and in the maintenance of vascular wall integrity. In mammals, actin exists in six isoforms expressed in a tissue-specific manner (Perrin and Ervasti, 2010). In vascular SMCs, α-actin (ACTA2) is the predominantly expressed actin isoform and the most abundant protein accounting for ~40% of the total cellular protein load (Fatigati and Murphy, 1984).

In humans, heterozygous *ACTA2* mutations predispose individuals to aortic aneurysm (Table 1). To date, ~40 mutations have been identified in the *ACTA2* gene. Missense mutations in *ACTA2* are the predominant genetic component of familial TAAD, accounting for 12–21% of all cases (Guo et al., 2007; Morisaki et al., 2009; Disabella et al., 2011; Renard et al., 2013). In most families the disease segregates as an autosomal dominant trait with variable penetrance and high clinical heterogeneity. Aortic tissue from patients carrying *ACTA2* mutations show an abnormal medial layer of the vessel wall with a disorganized structure indicating actin filament instability and/or abnormal filament assembly (Regalado et al., 2015) (Guo et al., 2007; Morisaki et al., 2009). Several studies have addressed these mutations to get an insight into how *ACTA2* mutations can cause TAAD. Carriers of R258C mutation show high penetrance and poor prognosis with a median life expectancy of ~35 years of age (Regalado et al., 2015). Using a baculoviral system Lu et al. could show that the R285C mutation in α-actin resulted in a less stable filament with increased sensitivity to cleavage by cofilin, a decreased rate of polymerization and a slower interaction with smooth muscle myosin leading to reduced force generation (Lu et al., 2015). In a later study by the same group, similar biochemical properties on actin function were obtained studying ACTA2 mutation R179H (Lu et al., 2016). Carriers of this mutation show early onset of disease with high penetrance and poor patient prognosis causing multisystemic smooth muscle dysfunction (Milewicz et al., 2010b; Munot et al., 2012; Georgescu et al., 2015; Regalado et al., 2015). More recently, Liu et al. developed a model system to study R258C-induced effects in a cellular context. Using patient-derived dermal fibroblasts the authors could demonstrate that mutated smooth muscle α-actin abrogated multiple cytoskeletal functions attributed to induction of wild type smooth muscle α-actin, including stress fiber formation, focal adhesions, matrix contraction, cellular migration, and filamentous to soluble actin ratio (Liu et al., 2017). Similar findings have been obtained by mutating the same arginine residue to a histidine, R285H. Using budding yeast as a model system, Malloy et al. showed that the yeast R258H actin produced abnormal cytoskeletal morphology and filament instability (Malloy et al., 2012). Moreover, a study on human ACTA2 mutations N117T and R118Q revealed mutation-specific effects on actin behavior suggesting that several individual mechanisms may contribute to the pathogenesis of familial TAAD (Bergeron et al., 2011).

**Table 1:** List of identified ACTA2 mutations with clinical and pathological characteristics.

**ACTA2 gene mutation**	**Actin polymerization**	**Vascular pathology**	**Clinical characteristics**
p.R149C		Aortic tissue: proteoglycan accumulation, loss and fragmentation of elastic fibers, focal loss of SMCs, SMC disarray, SMC hyperplasia in vasa vasorum (Guo et al., 2007; Disabella et al., 2011)	TAAD, Stroke, premature CAD (Guo et al., 2009), Livedo reticularis (Guo et al., 2007), iris cysts (Morisaki et al., 2009), iris flocculi (Guo et al., 2007; Disabella et al., 2011; Chamney et al., 2015)
p.R118Q	Perturbs ACTA2 filament assembly or stability (Guo et al., 2007), causes filament instability with faster disassembly rates and increased critical concentrations, hypersensitive to cofilin severing (Bergeron et al., 2011)	Coronary and epicardial artery: Stenosis of the vessel with increased SMC proliferation (Guo et al., 2009)	TAAD, Stroke, premature CAD (Guo et al., 2007, 2009)
p.T353N	Perturbs ACTA2 filament assembly or stability (Guo et al., 2007)	Aortic tissue: SMC hyperplasia in vasa vasorum (Guo et al., 2007)	TAAD (Guo et al., 2007, 2009)
p.R258C/H	Causes actin filament instability, increased susceptibility to severing by cofilin, higher affinity binding to profilin, perturbed interaction with smooth muscle myosin, decreased rate of polymerization (Malloy et al., 2012; Lu et al., 2015)	Aortic tissue: proteoglycan accumulation, loss and fragmentation of elastic fibers, areas with SMC loss, SMC disarray (Guo et al., 2007)	TAAD, premature stroke including Moyamoya disease (Guo et al., 2009), PDA (Guo et al., 2007)
p.R39H			TAAD, premature stroke, CAD (Guo et al., 2009)
p.R39C			TAAD (Hoffjan et al., 2011; Renard et al., 2013)
p.P72Q			TAAD (Guo et al., 2009)
p.N117T	Causes filament instability, with faster disassembly rates and increased critical concentrations, hyposensitive to severing by cofilin (Bergeron et al., 2011)		TAAD, Stroke (Guo et al., 2009)
p.Y135H			TAAD (Guo et al., 2009)
p.V154A			TAAD (Guo et al., 2009)
p.G160D			TAAD (Guo et al., 2009)
p.R185Q			TAAD, CAD (Guo et al., 2009)
p.R212Q			TAAD (Guo et al., 2009; Morisaki et al., 2009), premature stroke, CAD (Guo et al., 2009)
p.P245H			TAAD, Stroke (Guo et al., 2009)
p.I250L			TAAD, Stroke (Guo et al., 2009)
p.R292G		Stenosis of epicardial arteries, increased SMC proliferation (Guo et al., 2009)	TAAD (Guo et al., 2009)
p.T326N			TAAD, Stroke, CAD (Guo et al., 2009)
p.T353N			TAAD, Stroke, CAD (Guo et al., 2009)
p.R179H	Increased susceptibility to severing by cofilin, higher affinity binding to profilin, perturbed interaction with smooth muscle myosin, increased disassembly rate, binds less cooperatively to MRTFA (Lu et al., 2016)	Aortic tissue: fibroproliferative lesions in the intima, medial SMC proliferation and fragmentation of elastic fiber, proteoglycan accumulation, stenosis of vasa vasorum (Milewicz et al., 2010a) (Georgescu et al., 2015) Cerebral arteries: Intimal thickening, medial fibrosis, fragmented and thickened elastic laminae, SMC proliferation (Munot et al., 2012) (Georgescu et al., 2015)	Ascending aortic aneurysm, PDA cerebrovascular disease, fixed dilated pupils, hypotonic bladder, malrotation, hypoperistalsis of the gut and pulmonary hypertension, congenital mydriasis (Milewicz et al., 2010a; Al-Mohaissen et al., 2012; Munot et al., 2012; Logeswaran et al., 2017)
p.D82E		Aortic wall: Loss of SMCs (Disabella et al., 2011)	TAAD, Myopia (*n* = 2/2) (Disabella et al., 2011)
p.E243K		Aortic wall: Loss of SMCs (Disabella et al., 2011)	TAAD, Myopia (*n* = 2/2) (Disabella et al., 2011)
p.V45L			TAAD (Disabella et al., 2011)
IVS4+1G>A		Aortic wall: SMC hyperplasia in vasa vasorum, disarray of medial SMC (Disabella et al., 2011)	TAAD, Scoliosis *(n* = 5/8), Pes planus (*n* = 5/8), Livedo reticularis (*n* = 1/8), Iris flocculi (*n* = 1/8), Myopia (*n* = 2/8) (Disabella et al., 2011)
p.M49V			TAAD (Hoffjan et al., 2011; Renard et al., 2013)
p.G340R			TAAD (Hoffjan et al., 2011)
p.G152_T205del			TAAD (Morisaki et al., 2009)
p.Y145C (sporadic case)			TAAD (Morisaki et al., 2009)
p.D26Y		Aortic tissue: medial degeneration with loss and fragmentation of elastic fibers, disarray and loss of SMCs, accumulation of proteoglycan, SMCs hyperplasia of vasa vasorum (*n* = 2) (Yoo et al., 2010)	TAAD (Yoo et al., 2010)
p.R314X			TAAD (Renard et al., 2013)
p.S340CfxX25			TAAD (Renard et al., 2013)
p.G38R			TAAD (Renard et al., 2013)
p.H42N			TAAD (Renard et al., 2013)
p.Q61R			TAAD (Renard et al., 2013)
p.N117I			TAAD (Ke et al., 2016)
p.L348R			TAAD (Ke et al., 2016)
p.Y168N (sporadic case)			TAAD, BAV (*n* = 1/1) (Ke et al., 2016)
p.K328N		Aortic tissue: elastic fiber fragmentation, SMC disarray, adventitial fibrosis (Ware et al., 2014)	TAAD, congenital mydriasis (*n* = 2/2) (Ware et al., 2014)

*CAD, coronary artery disease; BAV, bicuspid valve; PDA, patent ductus arteriosus; p, protein reference sequence*.

It seems that various types of arteries respond differently to the underlying mutation contributing to a diverse pathology (Guo et al., 2009). ACTA2 mutations lead to dilation of larger vessels such as the aorta but occlusion of smaller arteries. The different response arteries display to a single gene mutation has been attributed to several factors including vascular SMCs lineage diversity, elastic vs. muscular arteries, and differences in mechanical forces on the vascular wall (Guo et al., 2009; Milewicz et al., 2010a).

In addition to aortic aneurysms, other features associated with subset of families with *ACTA2* mutations include cases with BAV and a predisposition for occlusive vascular diseases, including thrombotic stroke and coronary artery disease (Guo et al., 2007, 2009; Ke et al., 2016). The association of BAV with TAAD has been reported frequently suggesting that a common gene defect underlies this association (Edwards et al., 1978; Loscalzo et al., 2007). Included in the occlusive vascular diseases were cases with livedo reticularis, a skin rash caused by occlusion of dermal arteries and Moyamoya, a cerebrovascular disease characterized by progressive stenosis (Guo et al., 2007, 2009; Bergeron et al., 2011). Studies on tissue from affected individuals demonstrate an excessive proliferation of SMCs and myofibroblasts contributing to vascular occlusion (Guo et al., 2009; Milewicz et al., 2010a). This increase in SMC proliferation has been attributed to the role α-actin plays in regulating smooth muscle phenotype by shifting the F/G-actin ratio. It is well-established that when F-actin polymerization is inhibited the monomeric pool of G-actin is increased. The downstream effects of an increased pool of G-actin include retention of the actin-binding transcription factor, myocardin-related transcription factor (MRTF-A/B), in the cytosol. MRTF is a transcriptional co-factor that complexes with serum response factor (SRF) to drive expression of SMC-specific genes. As a consequence, an increase in G-actin may alter the phenotype of SMCs, from a highly contractile phenotype to a more proliferative phenotype. In a recent study, we have demonstrated that polymerization of actin filaments, and MRTF-dependent gene expression, is reduced in mildly dilated aortas from patients with stenotic tricuspid aortic valve (TAV) or BAV (Alajbegovic et al., 2016). This result suggests that altered actin polymerization may be an early event in the development of ascending aortic aneurysm and that the effect is not specific for BAV-associated disease.

Knockout of MRTF-B results in embryonic lethality associated with a spectrum of cardiovascular defects including aortic aneurysms (Oh et al., 2005; Li et al., 2012). The importance of MRTF-A/B for aneurysm formation in adult mice using smooth muscle-specific inducible double knockout has to our knowledge not been investigated. However, tamoxifen inducible, SMC-specific deletion of myocardin in mice leads to dilation of the thoracic aorta, dissection and rupture mimicking the pathology seen in TAAD patients (Huang et al., 2015). Myocardin is a muscle-restricted transcription factor, part of the myocardin family of transcriptional coactivators that, similar to MRTFs, promotes the expression of smooth muscle specific genes.

Further support of an important role of myocardin family co-activators for aneurysm formation is suggested by studies using smooth muscle specific knockout mice of intergrin-linked kinase (ILK) (Shen et al., 2011). ILK is a serine/threonine kinase with the main function to link extracellular matrix (ECM) via integrins to the actin cytoskeleton (Qian et al., 2005). SMC-conditional ILK mutant mice die around the perinatal period exhibiting defective morphogenetic development of the greater arteries including aneurysmal dilation of the thoracic aorta. Histological analysis revealed a profound vascular pathology of the arterial tunica media with changes in SMC phenotype, disruption of elastic lamellae, and a decreased actin polymerization (Shen et al., 2011). In agreement with a role of actin for nuclear translocation of MRTF, ILK deletion caused cytoplasmic retention of MRTF-A in aortic SMCs. In support of this study, conditional deletion of *Ilk* in neural crest cells results in aortic aneurysm and embryonic lethality (Arnold et al., 2013). *Ilk* mutant mice show defective differentiation of neural crest cells into SMCs and disorganization of actin stress fibers. Thus, these studies suggest that ILK regulates a signaling pathway involving actin polymerization that protects against aortic aneurysm. As such, *Ilk* mutant mice may prove helpful as animal models for additional insight into the pathogenesis of arterial aneurysms.

## Role of microRNAs in aneurysm development

MicroRNAs (miRNAs) are small (~22 nt) non-coding RNAs that are involved in post-transcriptional regulation of protein synthesis (Bartel, 2004). The biogenesis of miRNAs involve transcription by RNA polymerases, cleavage by endoribonucleases Drosha and Dicer, and incorporation into the RNA-induced silencing complex (RISC). The miRNAs then target 3′-UTR of mRNAs by binding with partial complementarity to the mRNA sequence. Perfect base pairing of the seed region (nucleotides 2–7 of the miRNA) to the mRNA is necessary for miRNA-dependent regulation. Binding of the RISC complex to mRNAs results in translational inhibition and in some cases mRNA degradation. In recent years, several miRNAs have been shown to be involved in the development of vascular disease states including aneurysms (Albinsson and Sward, 2013; Duggirala et al., 2015). Altered composition of ECM proteins in the vascular wall is one of the hallmarks of aortic aneurysms. Both fibroblasts and SMCs play important roles in matrix deposition and several studies have focused on the potential involvement of miRNAs in the regulation of ECM synthesis in these cell types.

The miR-29 family (miR-29a/b/c) stands out in its ability to target mRNAs encoding for ECM proteins, including collagens, elastin, and fibrillin-1 (van Rooij et al., 2008; Ekman et al., 2013; Maegdefessel et al., 2014). Pioneering work by Boon et al. demonstrated increased expression of miR-29b in two mouse models of aortic aneurysm (AngII-treated aged mice and Fibulin4(R/R) mice) and in early aortic dilation associated with BAV and TAV in humans (Boon et al., 2011). Importantly, AngII mediated dilation of mouse aorta was prevented using LNA-modified antisense oligonucleotide-mediated silencing of miR-29 (Boon et al., 2011). The therapeutic effect of miR-29 inhibition is consistent in other animal models of aneurysm formation (Maegdefessel et al., 2012b) (Zampetaki et al., 2014). However, in contrast with the study by Boon et al., additional studies have demonstrated reduced expression of miR-29 family members in human aortic aneurysms (Jones et al., 2011; Maegdefessel et al., 2012b). This discrepancy may depend on several factors including differences in tissue sampling (as discussed below) and the characteristics of the aortic dilation (Maegdefessel et al., 2014).

In a recently published study, we investigated differential miRNA expression in the convexity and concavity of the aortic arch of patients with mildly dilated aortae associated with either BAV or TAV. These samples were compared with biopsies from healthy donors. The convexity of the aortic wall is more disease-prone than the concavity, which may depend on differences in wall shear stress on the endothelial cells (Atkins et al., 2014). Comparison of these two regions may thus be important to understand the role of flow dynamics for the development of ascending aortic wall remodeling. We found that miR-29a/c was upregulated in the aortic concavity of dilated aorta associated with BAV (Albinsson et al., 2017). Further analysis revealed that miR-29a/c expression was reduced in the convexity compared to the concavity in BAV. A two-fold threshold was used which excluded minor changes in miRNA expression. However, it is interesting to note that both miR-29a (fold change: −1.96) and miR-29c (fold change: −1.43) were reduced in the convexity of BAV aorta compared to donor controls. Only minor differences were observed in miR-29b expression in either setting. These results propose that the discrepancy in miR-29 expression in biopsies from patients with BAV associated aortic dilation may depend on the localization of the obtained tissue sample. Similar to miR-29, miR-15 family members (miR-15, miR-16, miR-195, and miR-497) targets several ECM components (Ott et al., 2011). However, while *in vivo* administration of miR-195 inhibitor increases ECM production, this effect is not sufficient to prevent aneurysm formation (Zampetaki et al., 2014).

**Figure 1:**
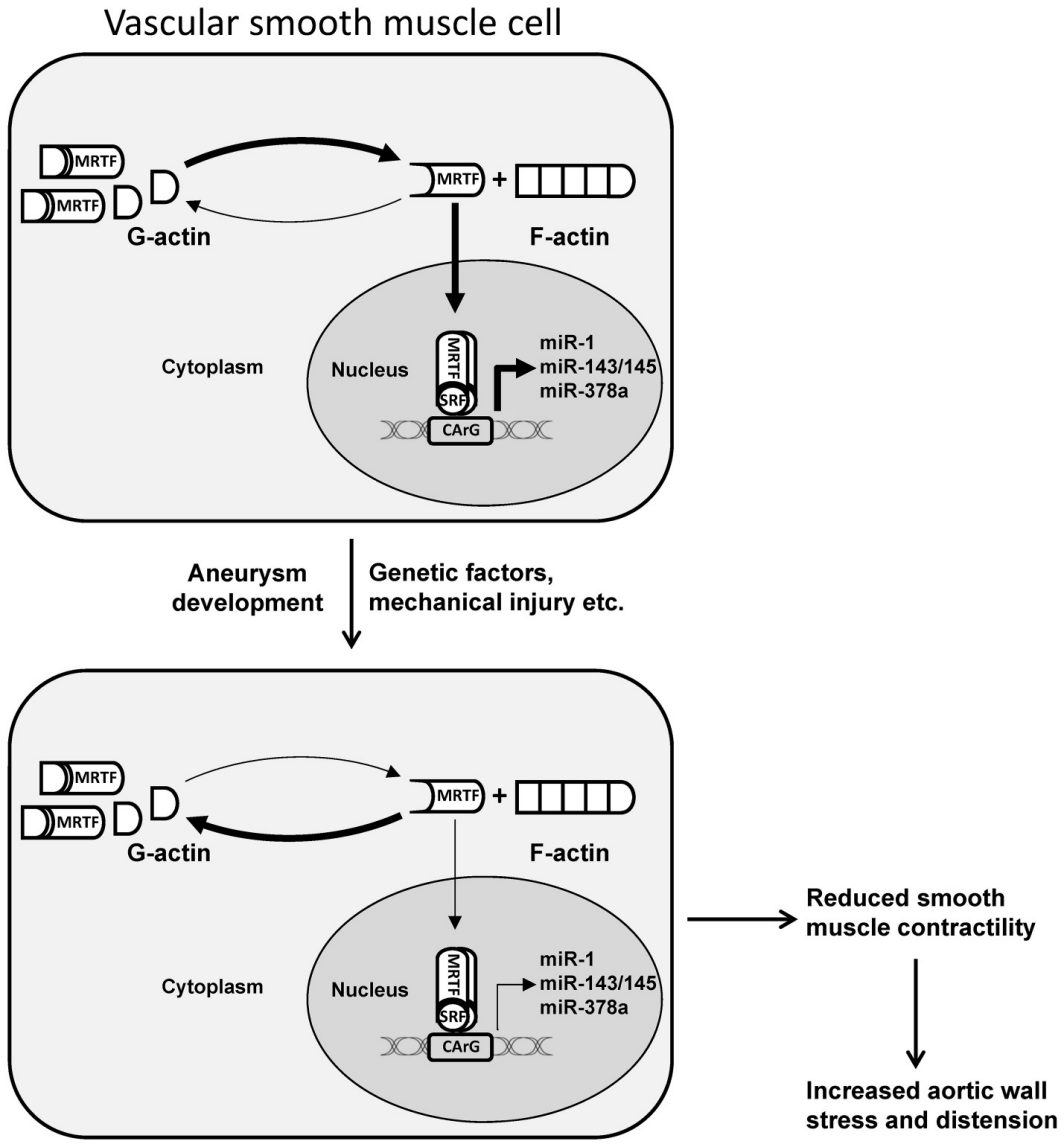
Schematic illustration of MRTF-dependent transcriptional regulation of miRNAs in dilated aorta. An increase in cytosolic or nuclear G-actin due to mutations in actin, mechanical injury, or other factors involved in aneurysm formation, results in decreased nuclear accumulation of MRTF and reduced transcription of miRNAs associated with contractile smooth muscle cells. In addition to other mechanisms, this can result in reduced smooth muscle contractility and increased aortic wall stress and distension. MRTF, myocardin related transcription factor; SRF, serum response factor. Adapted from Alajbegovic et al. (2016).

Although miR-29 appears to be particularly promising for therapeutic intervention, additional miRNAs have been demonstrated to be dysregulated in aortic aneurysms, including miR-21 (Maegdefessel et al., 2012a), miR-26 (Leeper et al., 2011), and the miR-143/145 cluster (Elia et al., 2009). In a recent study, we demonstrated that a group of smooth muscle miRNAs, including miR-143/145, miR-1, miR-378a, and miR-22, are regulated by actin dynamics via the actin sensitive transcription factor MRTF-A (Alajbegovic et al., 2016). With the exception of miR-22, these miRNAs were found to be highly enriched in muscle-containing tissues and downregulated in phenotypically modified SMCs. Interestingly, the levels of polymerized actin were reduced in biopsies of mildly dilated aorta (<4.5 cm) from patients with either stenotic TAV or BAV. Accordingly, the expression of the actin-regulated miRNAs was reduced in dilated aorta. These results point toward a role of actin polymerization and actin-sensitive transcription factors for the transcriptional control of miRNA expression in aortic aneurysm development (Figure 1). In support of this notion, miR-145 overexpression reduces the formation of AngII-induced AAA (Wu et al., 2016). The study by Wu et al. suggests that the effect of miR-145 involves reduced MMP2 activation. However, it is interesting to note that miR-145 has been shown to promote actin polymerization in smooth muscle cells (Xin et al., 2009; Albinsson et al., 2010), which may offer additional protection against aortic dilation.

Downregulation of miR-143/145 in the vascular wall has also been demonstrated in intracranial aneurysms (Jiang et al., 2013; Liu et al., 2014; Bekelis et al., 2016), suggesting that down-regulation of this miRNA cluster may be a general mechanism of aneurysm formation. Moreover, miR-21 is upregulated in both aortic (Maegdefessel et al., 2012a) and cerebral aneurysms (Bekelis et al., 2016). The increase in miR-21 and decrease in miR-143/145 clearly indicates reduced contractile differentiation of SMCs in the aneurysmal vascular wall. Although, phenotypic modulation of SMCs is likely to contribute to various vascular disease states, it is primarily an evolutionary conserved repair mechanism in response to vascular injury. Therefore, reversing this process can result in loss of endogenous protection against factors that promote aneurysm development. This is evident from results demonstrating that inhibition of miR-21 augments aortic dilation, while overexpression of miR-21 significantly reduced aortic dilation in an elastase-induced model of aortic aneurysm (Maegdefessel et al., 2012a). The effect of miR-21 overexpression is likely due to increased smooth muscle proliferation via decreased PTEN and increased Akt activation. This effect increases wall thickness and maintains wall stress at relatively low levels according to the Law of Laplace.

In summary, miRNAs are promising therapeutic targets against aneurysm formation. A common mechanism for the therapeutic effect of miRNAs appears to be to strengthen the vascular wall to better withstand mechanical forces of the blood pressure. This is accomplished by either increasing ECM production (miR-29 inhibitor), increasing smooth muscle growth (miR-21 mimic), or increasing smooth muscle contractile differentiation (miR-145 mimic).

## Summary

In summary, several lines of evidence support a role of actin polymerization in the development of aortic aneurysms. The effects of altered actin polymerization may be mediated directly, via loss of structural integrity, and/or via reduced contractility resulting in hampered resistance to tensile stretch exerted by the blood pressure. Furthermore, increasing evidence suggests that the regulation of myocardin family co-activators by actin polymerization is essential for protection against aneurysm development. This effect may be mediated in part via small non-coding RNAs. Further studies are warranted to elucidate the importance of actin regulated miRNAs for the development of arterial aneurysms. In addition, although changes in the expression of some miRNAs appear to be general for several types of aneurysms, our detailed analysis of miRNA expression in BAV vs. TAV-associated aortopathy has revealed differential miRNA signatures in these conditions. These findings need to be further explored to understand the importance of the specific differences in miRNA expression in BAV-associated aortic dilation.

## Author contributions

JH wrote introduction; AA wrote section on actin polymerization; SA wrote section on miRNAs, and summary. All authors contributed equally to this work.

### Conflict of interest statement

The authors declare that the research was conducted in the absence of any commercial or financial relationships that could be construed as a potential conflict of interest. The reviewer LP and handling Editor declared their shared affiliation, and the handling Editor states that the process met the standards of a fair and objective review.

## References

[B1] Alajbegovic A. Turczynska K. M. Hien T. T. Cidad P. Sward K. Hellstrand P.. (2016). Regulation of microRNA expression in vascular smooth muscle by mrtf-a and actin polymerization. Biochim. Biophys. Acta 1864, 1088–1098. 10.1016/j.bbamcr.2016.12.00 27939432

[B2] Albinsson S. Sward K. (2013). Targeting smooth muscle microRNAs for therapeutic benefit in vascular disease. Pharmacol. Res. 75, 28–36. 10.1016/j.phrs.2013.04.003 23611811

[B3] Albinsson S. Della Corte A. Alajbegovic A. Krawczyk K. K. Bancone C. Galderisi U.. (2017). Patients with bicuspid and tricuspid aortic valve exhibit distinct regional microRNA signatures in mildly dilated ascending aorta. Heart Vessels 32, 750–767. 10.1007/s00380-016-0942-7 28102444

[B4] Albinsson S. Suarez Y. Skoura A. Offermanns S. Miano J. M. Sessa W. C. (2010). MicroRNAs are necessary for vascular smooth muscle growth, differentiation, and function. Arterioscler. Thromb. Vasc. Biol. 30, 1118–1126. 10.1161/ATVBAHA.109.200873 20378849 PMC2880481

[B5] Al-Mohaissen M. Allanson J. E. O'Connor M. D. Veinot J. P. Brandys T. M. Maharajh G.. (2012). Brachial artery occlusion in a young adult with an *ACTA2* thoracic aortic aneurysm. Vasc. Med. 17, 326–329. 10.1177/1358863X12453973 22946110

[B6] Arnold T. D. Zang K. Vallejo-Illarramendi A. (2013). Deletion of integrin-linked kinase from neural crest cells in mice results in aortic aneurysms and embryonic lethality. Dis. Model. Mech. 6, 1205–1212. 10.1242/dmm.011866 23744273 PMC3759340

[B7] Atkins S. K. Cao K. Rajamannan N. M. Sucosky P. (2014). Bicuspid aortic valve hemodynamics induces abnormal medial remodeling in the convexity of porcine ascending aortas. Biomech. Model. Mechanobiol. 13, 1209–1225. 10.1007/s10237-014-0567-7 24599392

[B8] Bartel D. P. (2004). MicroRNAs: genomics, biogenesis, mechanism, and function. Cell 116, 281–297. 10.1016/S0092-8674(04)00045-5 14744438

[B9] Bekelis K. Kerley-Hamilton J. S. Teegarden A. Tomlinson C. R. Kuintzle R. Simmons N.. (2016). MicroRNA and gene expression changes in unruptured human cerebral aneurysms. J. Neurosurg. 125, 1390–1399. 10.3171/2015.11.JNS151841 26918470 PMC5001931

[B10] Bergeron S. E. Wedemeyer E. W. Lee R. Wen K. K. McKane M. Pierick A. R.. (2011). Allele-specific effects of thoracic aortic aneurysm and dissection alpha-smooth muscle actin mutations on actin function. J. Biol. Chem. 286, 11356–11369. 10.1074/jbc.M110.203174 21288906 PMC3064192

[B11] Biddinger A. Rocklin M. Coselli J. Milewicz D. M. (1997). Familial thoracic aortic dilatations and dissections: a case control study. J. Vasc. Surg. 25, 506–511. 10.1016/S0741-5214(97)70261-1 9081132

[B12] Boon R. A. Seeger T. Heydt S. Fischer A. Hergenreider E. Horrevoets A. J.. (2011). MicroRNA-29 in aortic dilation: implications for aneurysm formation. Circ. Res. 109, 1115–1119. 10.1161/CIRCRESAHA.111.255737 21903938

[B13] Chamney S. McGimpsey S. McConnell V. Willoughby C. E. (2015). Iris flocculi as an ocular marker of *ACTA2* mutation in familial thoracic aortic aneurysms and dissections. Ophthalmic Genet. 36, 86–88. 10.3109/13816810.2013.833634 24020716

[B14] Dietz H. C. Cutting G. R. Pyeritz R. E. Maslen C. L. Sakai L. Y. Corson G. M.. (1991). Marfan syndrome caused by a recurrent de novo missense mutation in the fibrillin gene. Nature 352, 337–339. 10.1038/352337a0 1852208

[B15] Disabella E. Grasso M. Gambarin F. I. Narula N. Dore R. Favalli V.. (2011). Risk of dissection in thoracic aneurysms associated with mutations of smooth muscle alpha-actin 2 (*ACTA2*). Heart 97, 321–326. 10.1136/hrt.2010.204388 21212136

[B16] Duggirala A. Delogu F. Angelini T. G. Smith T. Caputo M. Rajakaruna C.. (2015). Non coding rnas in aortic aneurysmal disease. Front. Genet. 6:125. 10.3389/fgene.2015.00125 25883602 PMC4381652

[B17] Edwards W. D. Leaf D. S. Edwards J. E. (1978). Dissecting aortic aneurysm associated with congenital bicuspid aortic valve. Circulation 57, 1022–1025. 10.1161/01.CIR.57.5.1022 639201

[B18] Ekman M. Bhattachariya A. Dahan D. Uvelius B. Albinsson S. Sward K. (2013). miR-29 repression in bladder outlet obstruction contributes to matrix remodeling and altered stiffness. PLoS ONE 8:e82308. 10.1371/journal.pone.0082308 24340017 PMC3858279

[B19] Elia L. Quintavalle M. Zhang J. Contu R. Cossu L. Latronico M. V.. (2009). The knockout of mir-143 and -145 alters smooth muscle cell maintenance and vascular homeostasis in mice: correlates with human disease. Cell Death Differ. 16, 1590–1598. 10.1038/cdd.2009.153 19816508 PMC3014107

[B20] Fatigati V. Murphy R. A. (1984). Actin and tropomyosin variants in smooth muscles. Dependence on tissue type. J. Biol. Chem. 259, 14383–14388. 6501298

[B21] Georgescu M. M. Pinho Mda C. Richardson T. E. Torrealba J. Buja L. M. Milewicz D. M.. (2015). The defining pathology of the new clinical and histopathologic entity *ACTA2*-related cerebrovascular disease. Acta Neuropathol. Commun. 3:81 10.1186/s40478-015-0262-7 26637293 PMC4670506

[B22] Guo D. C. Pannu H. Tran-Fadulu V. Papke C. L. Yu R. K. Avidan N.. (2007). Mutations in smooth muscle alpha-actin (*ACTA2*) lead to thoracic aortic aneurysms and dissections. Nat. Genet. 39, 1488–1493. 10.1038/ng.2007.6 17994018

[B23] Guo D. C. Papke C. L. Tran-Fadulu V. Regalado E. S. Avidan N. Johnson R. J.. (2009). Mutations in smooth muscle alpha-actin (*ACTA2*) cause coronary artery disease, stroke, and moyamoya disease, along with thoracic aortic disease. Am. J. Hum. Genet. 84, 617–627. 10.1016/j.ajhg.2009.04.007 19409525 PMC2680995

[B24] Hoffjan S. Waldmuller S. Blankenfeldt W. Kotting J. Gehle P. Binner P.. (2011). Three novel mutations in the *ACTA2* gene in german patients with thoracic aortic aneurysms and dissections. Eur. J. Hum. Genet. 19, 520–524. 10.1038/ejhg.2010.239 21248741 PMC3083620

[B25] Hoffman J. I. Kaplan S. (2002). The incidence of congenital heart disease. J. Am. Coll. Cardiol. 39, 1890–1900. 10.1016/S0735-1097(02)01886-7 12084585

[B26] Huang J. Wang T. Wright A. C. Yang J. Zhou S. Li L.. (2015). Myocardin is required for maintenance of vascular and visceral smooth muscle homeostasis during postnatal development. Proc. Natl. Acad. Sci. U.S.A. 112, 4447–4452. 10.1073/pnas.1420363112 25805819 PMC4394251

[B27] Jiang Y. Zhang M. He H. Chen J. Zeng H. Li J.. (2013). MicroRNA/mrna profiling and regulatory network of intracranial aneurysm. BMC Med. Genomics 6:36. 10.1186/1755-8794-6-36 24079748 PMC3849943

[B28] Jones J. A. Stroud R. E. O'Quinn E. C. Black L. E. Barth J. L. Elefteriades J. A.. (2011). Selective microRNA suppression in human thoracic aneurysms: relationship of miR-29a to aortic size and proteolytic induction. Circ. Cardiovasc. Genet. 4, 605–613. 10.1161/CIRCGENETICS.111.960419 22010139 PMC3246193

[B29] Ke T. Han M. Zhao M. Wang Q. K. Zhang H. Zhao Y.. (2016). Alpha-actin-2 mutations in chinese patients with a non-syndromatic thoracic aortic aneurysm. BMC Med. Genet. 17:45. 10.1186/s12881-016-0310-6 27431987 PMC4950238

[B30] Leeper N. J. Raiesdana A. Kojima Y. Chun H. J. Azuma J. Maegdefessel L.. (2011). MicroRNA-26a is a novel regulator of vascular smooth muscle cell function. J. Cell. Physiol. 226, 1035–1043. 10.1002/jcp.22422 20857419 PMC3108574

[B31] Li J. Bowens N. Cheng L. Zhu X. Chen M. Hannenhalli S.. (2012). Myocardin-like protein 2 regulates tgfbeta signaling in embryonic stem cells and the developing vasculature. Development 139, 3531–3542. 10.1242/dev.082222 22899851 PMC3436110

[B32] Liu D. Han L. Wu X. Yang X. Zhang Q. Jiang F. (2014). Genome-wide microRNA changes in human intracranial aneurysms. BMC Neurol. 14:188. 10.1186/s12883-014-0188-x 25300531 PMC4210474

[B33] Liu Z. Chang A. N. Grinnell F. Trybus K. M. Milewicz D. M. Stull J. T.. (2017). Vascular disease-causing mutation, smooth muscle alpha-actin r258c, dominantly suppresses functions of alpha-actin in human patient fibroblasts. Proc. Natl. Acad. Sci. U.S.A. 114, E5569–E5578. 10.1073/pnas.1703506114 28652363 PMC5514740

[B34] Loeys B. L. Schwarze U. Holm T. Callewaert B. L. Thomas G. H. Pannu H.. (2006). Aneurysm syndromes caused by mutations in the tgf-beta receptor. N. Engl. J. Med. 355, 788–798. 10.1056/NEJMoa055695 16928994

[B35] Logeswaran T. Friedburg C. Hofmann K. Akintuerk H. Biskup S. Graef M.. (2017). Two patients with the heterozygous r189h mutation in *ACTA2* and complex congenital heart defects expands the cardiac phenotype of multisystemic smooth muscle dysfunction syndrome. Am. J. Med. Genet. A 173, 959–965. 10.1002/ajmg.a.38102 28328125

[B36] Loscalzo M. L. Goh D. L. Loeys B. Kent K. C. Spevak P. J. Dietz H. C. (2007). Familial thoracic aortic dilation and bicommissural aortic valve: a prospective analysis of natural history and inheritance. Am. J. Med. Genet. A 143A, 1960–1967. 10.1002/ajmg.a.31872 17676603

[B37] Lu H. Fagnant P. M. Bookwalter C. S. Joel P. Trybus K. M. (2015). Vascular disease-causing mutation r258c in *ACTA2* disrupts actin dynamics and interaction with myosin. Proc. Natl. Acad. Sci. U.S.A. 112, E4168–E4177. 10.1073/pnas.1507587112 26153420 PMC4534267

[B38] Lu H. Fagnant P. M. Krementsova E. B. Trybus K. M. (2016). Severe molecular defects exhibited by the r179h mutation in human vascular smooth muscle alpha-actin. J. Biol. Chem. 291, 21729–21739. 10.1074/jbc.M116.744011 27551047 PMC5076841

[B39] Maegdefessel L. Azuma J. Tsao P. S. (2014). MicroRNA-29b regulation of abdominal aortic aneurysm development. Trends Cardiovasc. Med. 24, 1–6. 10.1016/j.tcm.2013.05.002 23871588 PMC3815986

[B40] Maegdefessel L. Azuma J. Toh R. Deng A. Merk D. R. Raiesdana A.. (2012a). MicroRNA-21 blocks abdominal aortic aneurysm development and nicotine-augmented expansion. Sci. Transl. Med. 4:122ra122. 10.1126/scitranslmed.3003441 22357537 PMC5753594

[B41] Maegdefessel L. Azuma J. Toh R. Merk D. R. Deng A. Chin J. T.. (2012b). Inhibition of microRNA-29b reduces murine abdominal aortic aneurysm development. J. Clin. Invest. 122, 497–506. 10.1172/JCI61598 22269326 PMC3266800

[B42] Malloy L. E. Wen K. K. Pierick A. R. Wedemeyer E. W. Bergeron S. E. Vanderpool N. D.. (2012). Thoracic aortic aneurysm (taad)-causing mutation in actin affects formin regulation of polymerization. J. Biol. Chem. 287, 28398–28408. 10.1074/jbc.M112.371914 22753406 PMC3436569

[B43] Milewicz D. M. Kwartler C. S. Papke C. L. Regalado E. S. Cao J. Reid A. J. (2010a). Genetic variants promoting smooth muscle cell proliferation can result in diffuse and diverse vascular diseases: evidence for a hyperplastic vasculomyopathy. Genet. Med. 12, 196–203. 10.1097/GIM.0b013e3181cdd687 20130469

[B44] Milewicz D. M. Ostergaard J. R. Ala-Kokko L. M. Khan N. Grange D. K. Mendoza-Londono R.. (2010b). *De novo ACTA2* mutation causes a novel syndrome of multisystemic smooth muscle dysfunction. Am. J. Med. Genet. A 152A, 2437–2443. 10.1002/ajmg.a.33657 20734336 PMC3573757

[B45] Morisaki H. Akutsu K. Ogino H. Kondo N. Yamanaka I. Tsutsumi Y. (2009). Mutation of *ACTA2* gene as an important cause of familial and nonfamilial nonsyndromatic thoracic aortic aneurysm and/or dissection (taad). Hum. Mutat. 30, 1406–1411. 10.1002/humu.21081 19639654

[B46] Morisaki T. Morisaki H. (2016). Genetics of hereditary large vessel diseases. J. Hum. Genet. 61, 21–26. 10.1038/jhg.2015.119 26446364

[B47] Munot P. Saunders D. E. Milewicz D. M. Regalado E. S. Ostergaard J. R. Braun K. P.. (2012). A novel distinctive cerebrovascular phenotype is associated with heterozygous arg179 *ACTA2* mutations. Brain 135, 2506–2514. 10.1093/brain/aws172 22831780 PMC3407424

[B48] Oh J. Richardson J. A. Olson E. N. (2005). Requirement of myocardin-related transcription factor-b for remodeling of branchial arch arteries and smooth muscle differentiation. Proc. Natl. Acad. Sci. U.S.A. 102, 15122–15127. 10.1073/pnas.0507346102 16204380 PMC1257726

[B49] Ott C. E. Grunhagen J. Jager M. Horbelt D. Schwill S. Kallenbach K.. (2011). MicroRNAs differentially expressed in postnatal aortic development downregulate elastin via 3′ utr and coding-sequence binding sites. PLoS ONE 6:e16250. 10.1371/journal.pone.0016250 21305018 PMC3031556

[B50] Perrin B. J. Ervasti J. M. (2010). The actin gene family: function follows isoform. Cytoskeleton 67, 630–634. 10.1002/cm.20475 20737541 PMC2949686

[B51] Prakash S. K. Bosse Y. Muehlschlegel J. D. Michelena H. I. Limongelli G. Della Corte A.. (2014). A roadmap to investigate the genetic basis of bicuspid aortic valve and its complications: insights from the international bavcon (bicuspid aortic valve consortium). J. Am. Coll. Cardiol. 64, 832–839. 10.1016/j.jacc.2014.04.073 25145529 PMC4485610

[B52] Qian Y. Zhong X. Flynn D. C. Zheng J. Z. Qiao M. Wu C.. (2005). Ilk mediates actin filament rearrangements and cell migration and invasion through pi3k/akt/rac1 signaling. Oncogene 24, 3154–3165. 10.1038/sj.onc.1208525 15735674

[B53] Regalado E. S. Guo D. C. Prakash S. Bensend T. A. Flynn K. Estrera A.. (2015). Aortic disease presentation and outcome associated with *ACTA2* mutations. Circ. Cardiovasc. Genet. 8, 457–464. 10.1161/CIRCGENETICS.114.000943 25759435 PMC4601641

[B54] Renard M. Callewaert B. Baetens M. Campens L. MacDermot K. Fryns J. P.. (2013). Novel myh11 and *ACTA2* mutations reveal a role for enhanced tgfbeta signaling in ftaad. Int. J. Cardiol. 165, 314–321. 10.1016/j.ijcard.2011.08.079 21937134 PMC3253210

[B55] Roth G. A. Huffman M. D. Moran A. E. Feigin V. Mensah G. A. Naghavi M.. (2015). Global and regional patterns in cardiovascular mortality from 1990 to 2013. Circulation 132, 1667–1678. 10.1161/CIRCULATIONAHA.114.008720 26503749

[B56] Shen D. Li J. Lepore J. J. Anderson T. J. Sinha S. Lin A. Y.. (2011). Aortic aneurysm generation in mice with targeted deletion of integrin-linked kinase in vascular smooth muscle cells. Circ. Res. 109, 616–628. 10.1161/CIRCRESAHA.110.239343 21778429 PMC3351207

[B57] Siu S. C. Silversides C. K. (2010). Bicuspid aortic valve disease. J. Am. Coll. Cardiol. 55, 2789–2800. 10.1016/j.jacc.2009.12.068 20579534

[B58] van Rooij E. Sutherland L. B. Thatcher J. E. DiMaio J. M. Naseem R. H. Marshall W. S.. (2008). Dysregulation of microRNAs after myocardial infarction reveals a role of miR-29 in cardiac fibrosis. Proc. Natl. Acad. Sci. U.S.A. 105, 13027–13032. 10.1073/pnas.0805038105 18723672 PMC2529064

[B59] Ware S. M. Shikany A. Landis B. J. James J. F. Hinton R. B. (2014). Twins with progressive thoracic aortic aneurysm, recurrent dissection and *ACTA2* mutation. Pediatrics 134, e1218–e1223. 10.1542/peds.2013-2503 25225139

[B60] Wu J. Wang J. Li X. Liu X. Yu X. Tian Y. (2016). MicroRNA-145 mediates the formation of angiotensin ii-induced murine abdominal aortic aneurysm. Heart Lung Circ. 6, 619–626. 10.1016/j.hlc.2016.10.009 27956160

[B61] Xin M. Small E. M. Sutherland L. B. Qi X. McAnally J. Plato C. F.. (2009). MicroRNAs miR-143 and miR-145 modulate cytoskeletal dynamics and responsiveness of smooth muscle cells to injury. Genes Dev. 23, 2166–2178. 10.1101/gad.1842409 19720868 PMC2751981

[B62] Yoo E. H. Choi S. H. Jang S. Y. Suh Y. L. Lee I. Song J. K.. (2010). Clinical, pathological, and genetic analysis of a korean family with thoracic aortic aneurysms and dissections carrying a novel asp26tyr mutation. Ann. Clin. Lab. Sci. 40, 278–284. 20689142

[B63] Zampetaki A. Attia R. Mayr U. Gomes R. S. Phinikaridou A. Yin X.. (2014). Role of miR-195 in aortic aneurysmal disease. Circ. Res. 115, 857–866. 10.1161/CIRCRESAHA.115.304361 25201911

[B64] Zhu L. Vranckx R. Khau Van Kien P. Lalande A. Boisset N. Mathieu F.. (2006). Mutations in myosin heavy chain 11 cause a syndrome associating thoracic aortic aneurysm/aortic dissection and patent ductus arteriosus. Nat. Genet. 38, 343–349. 10.1038/ng1721 16444274

